# Children and youth with non-traumatic brain injury: a population based perspective

**DOI:** 10.1186/s12883-016-0631-2

**Published:** 2016-07-20

**Authors:** Vincy Chan, Jason D. Pole, Michelle Keightley, Robert E. Mann, Angela Colantonio

**Affiliations:** Toronto Rehabilitation Institute, University Health Network, 550 University Avenue, Toronto, ON M5G 2A2 Canada; Rehabilitation Sciences Institute, University of Toronto, 500 University Avenue, Toronto, ON M5G 1 V7 Canada; Pediatric Oncology Group of Ontario, 480 University Avenue, Toronto, ON M5G 1 V2 Canada; Dalla Lana School of Public Health, University of Toronto, 155 College Street, Toronto, ON M5T 3 M7 Canada; Holland Bloorview Kids Rehabilitation Hospital, 150 Kilgour Road, Toronto, ON M5G 1R8 Canada; Centre for Addiction and Mental Health, 33 Russell Street, Toronto, ON M5S 3M1 Canada

**Keywords:** Non-traumatic brain injury, International Classification of Diseases, Pediatrics

## Abstract

**Background:**

Children and youth with non-traumatic brain injury (nTBI) are often overlooked in regard to the need for post-injury health services. This study provided population-based data on their burden on healthcare services, including data by subtypes of nTBI, to provide the foundation for future research to inform resource allocation and healthcare planning for this population.

**Methods:**

A retrospective cohort study design was used. Children and youth with nTBI in population-based healthcare data were identified using International Classification of Diseases Version 10 codes. The rate of nTBI episodes of care, demographic and clinical characteristics, and discharge destinations from acute care and by type of nTBI were identified.

**Results:**

The rate of pediatric nTBI episodes of care was 82.3 per 100,000 (*N* = 17,977); the average stay in acute care was 13.4 days (SD = 25.6 days) and 35 % were in intensive care units. Approximately 15 % were transferred to another inpatient setting and 6 % died in acute care. By subtypes of nTBI, the highest rates were among those with a diagnosis of toxic effect of substances (22.7 per 100,000), brain tumours (18.4 per 100,000), and meningitis (15.4 per 100,000). Clinical characteristics and discharge destinations from the acute care setting varied by subtype of nTBI; the proportion of patients that spent at least one day in intensive care units and the proportion discharged home ranged from 25.9 % to 58.2 % and from 50.6 % to 76.4 %, respectively.

**Conclusions:**

Children and youth with nTBI currently put an increased demand on the healthcare system. Active surveillance of and in-depth research on nTBI, including subtypes of nTBI, is needed to ensure that timely, appropriate, and targeted care is available for this pediatric population.

## Background

Acquired brain injury (ABI) is “an insult to the brain that affects its structure or function, resulting in impairments of cognition, communication, physical function, or psychosocial behavior” and “does not include brain injuries that are congenital, degenerative, or induced by birth trauma” [1]. To date, much attention has been placed on brain injuries from traumatic causes (i.e., a traumatic brain injury, TBI). However, it is important to recognize that brain injuries from non-traumatic causes (i.e., non-traumatic brain injury, nTBI) can also result in negative and long-term consequences [2]. These nTBI include anoxia, vascular insults, toxic effect of substances, brain tumours, meningitis, metabolic encephalopathy, encephalitis, and other brain disorders [1].

Comprehensive information on the health service use among the nTBI population is currently only available for adult and older adult populations [3–12]. These studies found that the direct cost of healthcare services for nTBI was higher than that of TBI ($120.7 vs. $368.7 million) [6]. In Ontario, Canada, between the fiscal years of 2003/04 and 2009/10, approximately 10 % of nTBI cases in the acute care setting were among patients aged 18 years and under [9]. An understanding of this pediatric population is important because healthcare, in particular rehabilitation, for the pediatric nTBI population often occurs in similar or identical programs and facilities as those of TBI [13, 14]. However as a group, nTBI are often overlooked with regard to the potential for long lasting sequelae and the need for healthcare services post-injury [15].

Equally important is the need to understand each subtype of nTBI, as nTBI includes diverse health conditions that may require a targeted approach to resource allocation and healthcare planning. Unfortunately, there is currently a lack of population based research and data on subtypes of pediatric nTBI with regard to their healthcare use. For example, even though primary brain tumours are the leading causes of cancer death in children and youth aged 19 years and under [16, 17], there lacks a general epidemiological profile and healthcare utilization information for this population. Research on childhood cancer indicate that there is no clear end to the duration of healthcare need among survivors and these patients continue to use substantial healthcare resources that are not accounted for in resource allocation [18]. As such, children and youth with brain tumors may be candidates for increased healthcare use. Similarly, a systematic review of out-of-hospital pediatric cardiac arrest and drowning, which are common causes of anoxic brain injuries [19, 20], showed that, while less than 7 % of children with cardiac arrest survive to hospital discharge, only 2.2 % survive without neurological sequelae. A fifth of patients with anoxic brain injuries due to near drowning survive to hospital discharge, but survival can be as high as 80 % if timely and appropriate actions are taken [19]. This suggests that this population is also likely to be users of acute-care services and in particular, post-hospitalization services such as homecare or rehabilitation. NTBI from infectious causes (e.g., meningitis, encephalitis) are most common among pediatric age groups, with persisting sequelae often reported, including fatigue, epilepsy, and impairments in cognition, memory, and motor [21–24]. As such, despite the diversity of causes of nTBI, the neurological sequelae and need for health services are evident across subtypes of nTBI.

The objectives of this study are to provide population-based information on (1) the burden of nTBI on healthcare services by identifying the rate of nTBI episodes of care in the province of Ontario in Canada and (2) the demographic and clinical characteristics and discharge destinations of hospitalized children and youth aged 19 years and under with nTBI. Recognizing the diverse conditions captured as nTBI, this paper additionally aimed to provide data on subtypes of nTBI that can be used to guide additional research on each type of nTBI. An understanding of the epidemiological profile and healthcare use of this group of individuals can greatly assist to appropriately, adequately, and effectively plan healthcare services for this population to ensure that their needs are met. This study is a first step towards a greater understanding of children and youth with nTBI and the patterns of their healthcare use.

## Methods

The Canadian Institute for Health Information (CIHI) National Ambulatory Care Reporting System (NACRS) and the Discharge Abstract Database (DAD) were used. The NACRS is a mandated data collection system that collects emergency department (ED) and ambulatory care data [25]. A reabstraction study of the NACRS data that compared 7,500 charts from 15 hospitals in Ontario from 2004 to 2005 indicated up to 80 % agreement for International Classification of Diseases Version 10 (ICD-10) codes for patients’ main problem (i.e., the health condition responsible for the patient’s visit, requiring evaluation and/or treatment/management) [25]. The DAD contains demographic and clinical information on all hospital admissions and discharges, including transfers and deaths, using standard diagnosis and procedure/intervention codes [26]. A reabstraction study of the DAD found high sensitivity and near perfect specificity for demographic variables and moderate to substantial agreement for diagnoses (kappa value 0.41 to 0.80) [27]. As residents of Ontario have universal access to ED and hospital-based care, these data sources allow for the identification of all children and youth with a nTBI diagnoses in the ED and/or acute care setting during our study period.

NTBI was categorized by the presence of specified ICD-10 codes in any of the 10 diagnosis fields in the NACRS and the 25 diagnosis fields in the DAD. These ICD-10 codes represent conditions that are captured as a nTBI as defined by the Commission on Accreditation of Rehabilitation Facilities (CARF) International [1] and through stakeholder consultation in Ontario, Canada. This included members from the Ontario Ministry of Health and Long-Term Care, Ontario Agency for Protection and Promotion, SMARTRISK, and the Ministry of Transportation to advise on the creation of a neurotrauma surveillance system to inform prevention in the province of Ontario [28] (Table 1). Stroke captured in national studies were excluded from our case definition for vascular insults to reflect current research and clinical practice in Ontario, Canada. For example, various national rehabilitation information systems [29, 30] and centres [31, 32] classify stroke separately from nTBI and some definitions of nTBI include vascular conditions that are not captured in major stroke studies [33]. As such, these vascular insults were included in this study. All subtypes of nTBI across the 10 diagnosis fields in the NACRS and 25 diagnosis fields in the DAD were counted.

**Table 1:** International Classification of Diseases version 10 (ICD-10) case definitions for non-traumatic brain injury

Type of Non-Traumatic brain injury	ICD-10 Codes
Toxic effect of substances	T40.5, T42.6, T51, T56, T57.0, T57.2, T57.3, T58, T64, T65.0
Anoxia	G93.1, T71, T75.1, R09.0
Vascular insults (not captured in other national studies of stroke)	I62.0, I62.9
Brain tumours	C70, C71, C79.3, C79.4, D32.0, D33.0, D33.1, D33.2, D33.3, D42.0, D43, D43.2
Encephalitis	A81.1, A83.0, A83.2, A86.0, B00.4, B01.1, B02.0, B05.0, B94.1, G04.0, G04.2, G04.8, G04.9, G05, G09
Metabolic encephalopathy	E10.0, E11.0, E13.0, E14.0, E15, G92, G93.4
Meningitis	A87, B01.0, B37.5, G00, G01, G02, G03
Other brain disorders	G91.0, G91.1, G91.2, G93.2, G93.5, G93.6, G93.8, G93.9, G99.8, R29.1

Demographic variables included age and sex. Children and youth aged 19 years and under were categorized into five-year age groups [0 to 4 years (infants), 5 to 9 years (children), 10 to 14 years (youth), and 15 to 19 years (adolescents)] consistent with categories commonly used in the ABI literature [34], Statistics Canada [35], and the World Health Organization [36].

Clinical variables included the Charlson Comorbidity Index, length of stay (LOS) in acute care, and special care days. The Charlson Comorbidity Index is widely accepted as a useful tool for measuring comorbidity disease status, has been shown to have a consistent correlation to in-hospital mortality, and is used to assess the severity of comorbid health conditions [37, 38]. The score is derived using the sum of the standard weights (1 to 6) that are assigned for each condition in the index [39]. As most patients identified in this study have few Charlson comorbidities, the categories of 0 – 1 and 2+ were used to avoid the risk of re-identification of any patients in the acute care setting. LOS in acute care was defined as the number of days between admission and discharge. Special care days were defined as the cumulative number of days spent in all intensive care units.

Discharge disposition from acute care included death in acute care, home, home with support services (e.g., homecare, home making supportive housing), inpatient rehabilitation, complex continuing care (CCC; e.g., chronic care facility), long term care (LTC; e.g., nursing home), and transferred to another inpatient setting.

NTBI episodes of care between fiscal years 2003/04 and 2009/10 were used to determine the number and rate of healthcare utilization, which has been shown to provide a more accurate description of the utilization of healthcare services compared to data on just hospitalizations [40]. This was accomplished by linking the DAD to the NACRS via an unique encoded identifier, which is complete for all cases in the NACRS and the DAD and ensured that each episode was only captured once. The number of nTBI episodes of care by age group, sex, and type of nTBI were identified. The rate of nTBI episodes of care per 100,000 children and youth in Ontario, Canada, was calculated by dividing the total number of nTBI episodes by the population counts for the specific age group and sex in Ontario, Canada during the fiscal year of study. These numbers indicate the total number of nTBI episodes of care for every 100,000 children and youth in Ontario, Canada, during the study period.

A patient-level analysis of the patient’s initial hospitalization for a nTBI between 2004/05 and 2009/10 was used to examine patient and clinical characteristics, and discharge destination from acute care. This was chosen to distinguish between a readmissions profile, which may differ from the initial admission. Patients included in this analysis must be initial hospitalizations between fiscal years 2003/04 and 2009/10. A look-back window of at least one year was used to ensure that patients included were the initial hospitalization record between fiscal years 2003/04 and 2009/10. Due to the lack of data to look back one year for fiscal year 2003/04, this fiscal year was eliminated from the patient level analysis. This ensured that patients identified between fiscal years 2004/05 and 2009/10 were index hospitalizations during this study period.

## Results

**Fig. 1:**
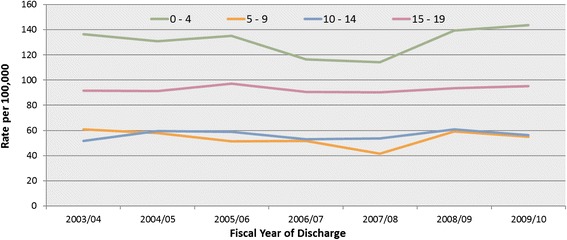
nTBI episodes of care by age groups and fiscal year of discharge

Between fiscal years 2003/04 and 2009/10, there were 17,977 nTBI episodes of care (82.3 per 100,000 children and youth 19 years and under in Ontario, Canada). Males had a higher rate (87.2 per 100,000) compared to females (77.2 per 100,000). By age group, the highest rates were among infants aged 0 to 4 years (130.8 per 100,000), followed by adolescents aged 15 to 19 years (92.9 per 100,000), youth aged 10 to 14 years (56.1 per 100,000), and children aged 5 to 9 years (54.1 per 100,000) (Table 2, Fig. 1).

**Table 2:** nTBI episodes of care (n and rate per 100,000 children and youth aged 19 years and under) in Ontario between fiscal years 2003/04 and 2009/10 by age, fiscal year of discharge, and sex

	Overall			0 – 4			5 – 9			10 – 14			15 – 19		
	Overall n rate	Male n rate	Female n rate	Overall n rate	Male n rate	Female n rate	Overall n rate	Male n rate	Female n rate	Overall n rate	Male n rate	Female n rate	Overall n rate	Male n rate	Female n rate
2003/04	2591 82.7	1437 89.7	1154 75.5	932 136.3	553 158.4	379 113.3	477 61.0	242 60.7	235 61.3	432 51.6	252 58.9	171 41.8	759 91.6	390 91.5	369 91.6
2004/05	2606 83.2	1406 87.7	1200 78.5	893 131.0	522 149.4	371 111.7	445 58.0	247 63.2	198 52.7	500 59.4	267 62.1	233 56.6	768 91.3	370 85.5	398 97.3
2005/06	2635 84.2	1455 90.8	1180 77.3	922 135.1	539 153.8	383 115.4	385 51.4	226 59.1	159 43.3	494 58.9	278 64.9	216 52.6	834 97.2	412 93.5	422 101.1
2006/07	2419 77.3	1327 82.8	1092 71.6	808 116.4	441 123.6	367 108.8	379 51.8	242 64.6	137 38.4	440 53.0	228 53.9	212 52.2	792 90.7	416 92.9	376 88.4
2007/08	2328 74.7	1269 79.5	1059 69.7	794 114.1	433 120.9	361 106.8	300 41.6	176 47.5	124 35.3	439 53.8	233 55.9	206 51.6	795 90.3	427 94.8	368 85.6
2008/09	2717 87.5	1453 91.3	1264 83.4	975 139.5	578 160.8	397 116.9	424 59.1	240 65.2	184 52.7	488 60.8	245 59.8	243 62.0	830 93.6	390 86.1	440 101.4
2009/10	2681 86.3	1404 88.3	1277 84.2	986 143.5	561 159.1	425 127.1	399 55.1	228 61.6	171 48.3	459 56.2	218 52.3	241 60.2	837 95.3	397 88.1	440 102.9
2003/04 – 2009/10	17977 82.3	9751 87.2	8226 77.2	6310 130.8	3627 146.5	2683 114.3	2809 54.1	1601 60.3	1208 47.6	3243 56.1	1721 58.3	1522 53.8	5615 92.9	2802 90.4	2813 95.5

**Fig. 2:**
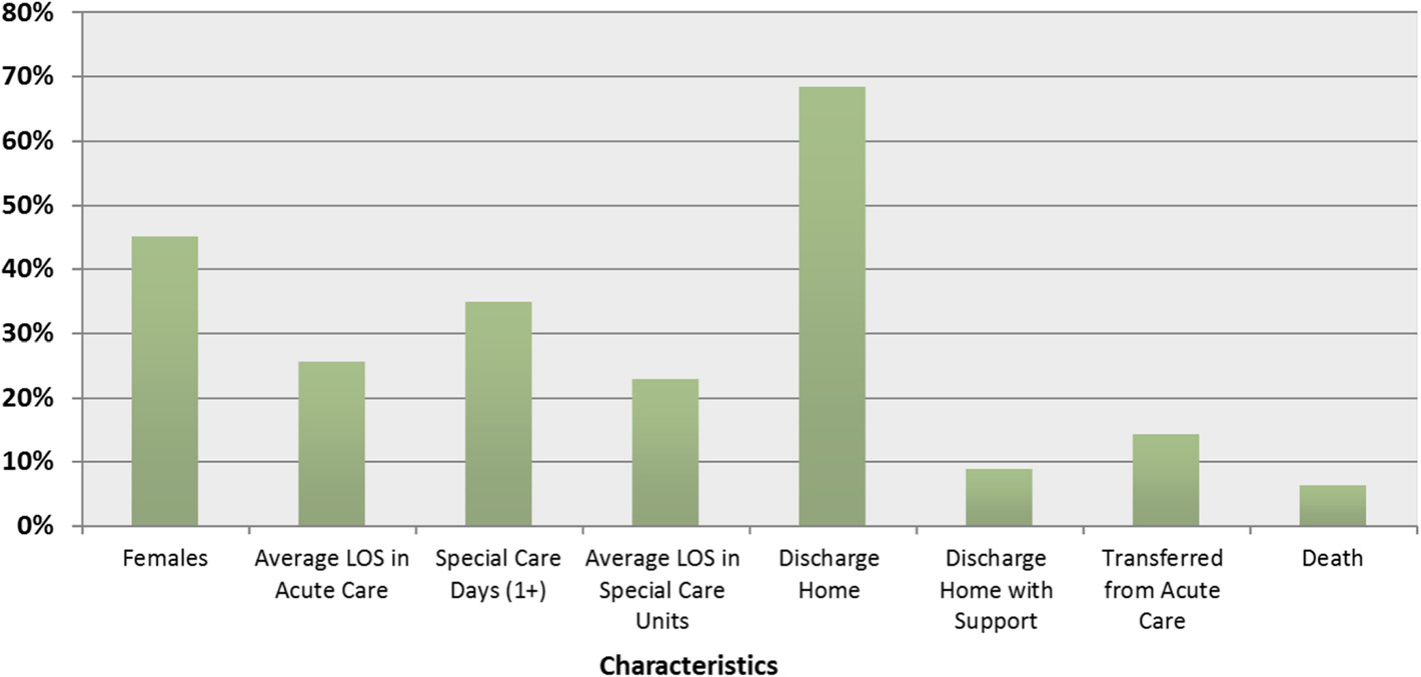
Patient characteristics by type of nTBI

Patient level analyses showed that there were 6,102 patients with nTBI between fiscal years 2004/05 and 2009/10. The average LOS in acute care was 13.4 days (SD = 25.6 days) and among those with at least one special care days (35 %), the average stay in intensive care units was 12.5 days (SD = 22.9 days). Approximately 12 % of patients had a Charlson Comorbidity Index Score of 2 or higher. Most of the patients (77 %) were discharged home from acute care, of which 9 % were discharged home with support services. Approximately 14 % of patients were transferred to another inpatient setting, less than 1 % to inpatient rehabilitation, 1 % to CCC or LTC, and 6 % died in acute care (Table 3, Fig. 2).

**Table 3:** Patient characteristics among children and youth with a nTBI diagnostic code in acute care in Ontario between fiscal years 2004/05 and 2009/10 by type of nTBI

Characteristics	Overall^a^		Toxic effect of substances		Anoxia		Vascular insults		Brain tumours		Encephalitis		Metabolic encephalopathy		Meningitis		Other brain disorders	
	*N*	Col %	*N*	Col %	*N*	Col %	*N*	Col %	*N*	Col %	*N*	Col %	*N*	Col %	*N*	Col %	*N*	Col %
Overall	6102	100	713	100	903	100	182	100	745	100	438	100	325	100	2094	100	1077	100
Age Groups																		
0 – 4	2713	44.5	59	8.3	530	58.7	108	59.3	174	23.4	177	40.4	117	36.0	1301	62.1	387	35.9
5 – 9	819	13.4	13	1.8	123	13.6	18	9.9	182	24.4	98	22.4	55	16.9	225	10.7	165	15.3
10 – 14	872	15.9	97	13.6	99	11.0	31	17.0	190	25.5	77	17.6	70	21.5	220	10.5	249	23.1
15 – 19	1598	26.2	544	76.3	151	16.7	25	13.7	199	26.7	86	19.6	83	25.5	303	14.5	276	25.6
Sex																		
Males	3358	55.0	321	45.0	539	59.7	114	62.6	410	55.0	224	51.1	172	52.9	1163	56.8	595	55.3
Females	2744	45.0	392	55.0	364	40.3	68	37.4	335	45.0	214	48.9	153	47.1	886	43.2	482	44.8
Length of Stay (Days)																		
Average Length of Stay (Mean, SD)	13.4 (25.6)		4.0 (6.4)		15.2 (36.4)		26.7 (44.1)		14.0 (22.4)		16.7 (23.9)		20.2 (41.0)		13.8 (21.6)		16.0 (25.9)	
1 – 2	1278	20.9	390	54.7	287	31.8	16	8.8	108	14.5	58	13.2	48	14.8	209	10.2	195	18.1
3 – 5	1835	30.1	224	31.4	234	25.9	40	22.0	195	26.2	107	24.4	73	22.5	699	34.1	326	30.3
6 – 11	1296	21.2	61	8.6	153	16.9	42	23.1	217	29.1	109	24.9	78	24.0	482	23.5	212	19.7
12+	1693	27.8	38	5.3	229	25.4	84	46.2	225	30.2	164	37.4	126	38.8	659	32.2	344	31.9
Charlson Comorbidity Index Score																		
0 – 1	5355	87.8	NR	.	855	94.7	143	78.6	241	32.4	411	93.8	278	85.5	2005	97.9	908	84.3
2+	747	12.2	<5	.	48	5.3	39	21.4	504	67.7	27	6.2	47	14.5	44	2.1	169	15.7
Special Care Days																		
Average Number of Days (Mean, SD)	12.5 (22.9)		1.9 (2.4)		14.6 (28.8)		16.1 (22.5)		4.7 (12.8)		13.7 (18.0)		10.2 (15.0)		21.6 (27.8)		11.4 (20.4)	
None	3970	65.1	475	66.6	522	57.8	76	41.8	377	50.6	327	74.7	181	55.7	1519	74.1	591	54.9
1 – 2	865	14.2	197	27.6	117	13.0	21	11.5	247	33.2	25	5.7	35	10.8	114	5.6	185	17.2
3 – 5	370	6.1	36	5.1	70	7.8	19	10.4	69	9.3	25	5.7	43	13.2	70	3.4	94	8.7
6 – 11	315	5.2	<5	.	70	7.8	NR	.	22	3.0	20	4.6	34	10.5	97	4.7	81	7.5
12+	582	9.5	<5	.	124	13.7	NR	.	30	4.0	41	9.4	32	9.9	249	12.2	126	11.7
Discharge Disposition																		
Home	4175	68.4	545	76.4	554	61.4	92	50.6	487	65.4	259	59.1	206	63.4	1490	72.7	653	60.6
Home with Support	544	8.9	11	1.5	73	8.1	35	19.2	132	17.7	71	16.2	45	13.9	131	6.4	121	11.2
Rehabilitation	47	0.8	<5	.	9	1.0	<5	.	9	1.2	8	1.8	<5	.	<5	.	15	1.4
Complex Continuing Care or Long Term Care	81	1.3	0	0	14	1.6	7	3.9	13	1.7	15	3.4	NR	.	NR	.	32	2.9
Transferred	870	14.3	151	21.2	78	8.6	24	13.2	80	10.7	76	17.4	28	8.6	372	18.2	99	9.2
Death	385	6.3	<5	.	175	19.4	NR	.	24	3.2	9	2.1	32	9.9	44	2.2	157	14.6

Note: ^a^All multiple nTBI diagnoses were counted for and as such, the overall N will not add up

NR = not reportable due to small cell sizes

**Fig. 3:**
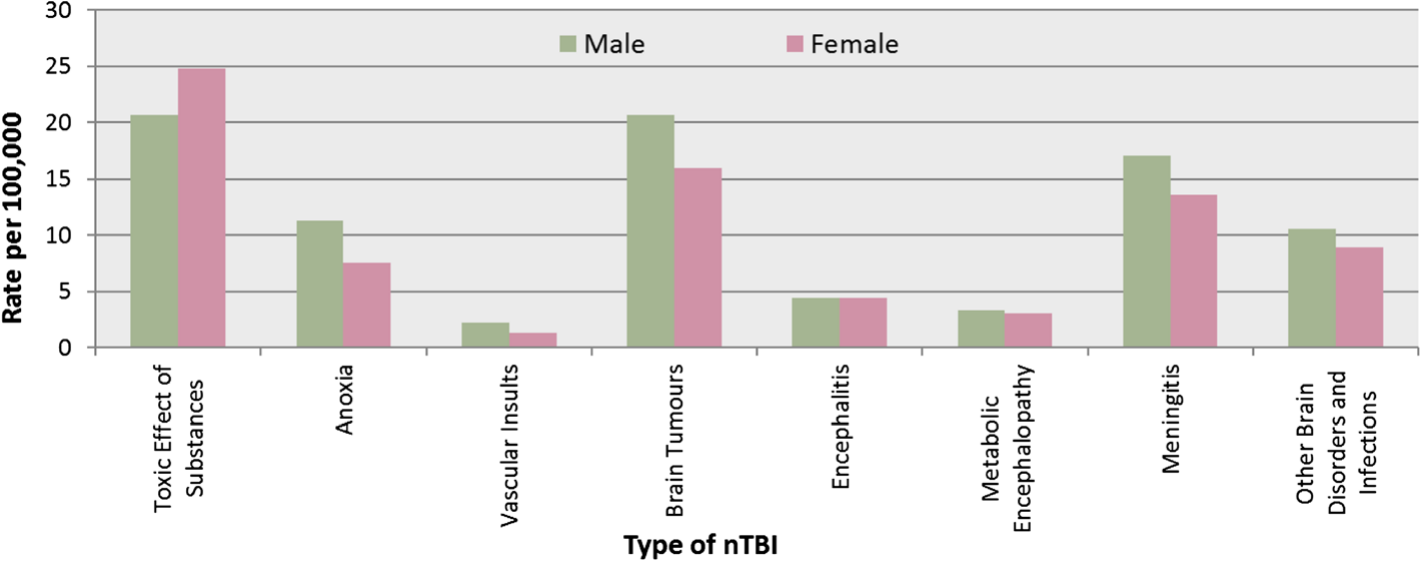
nTBI episodes of care by type of nTBI and sex

**Fig. 4:**
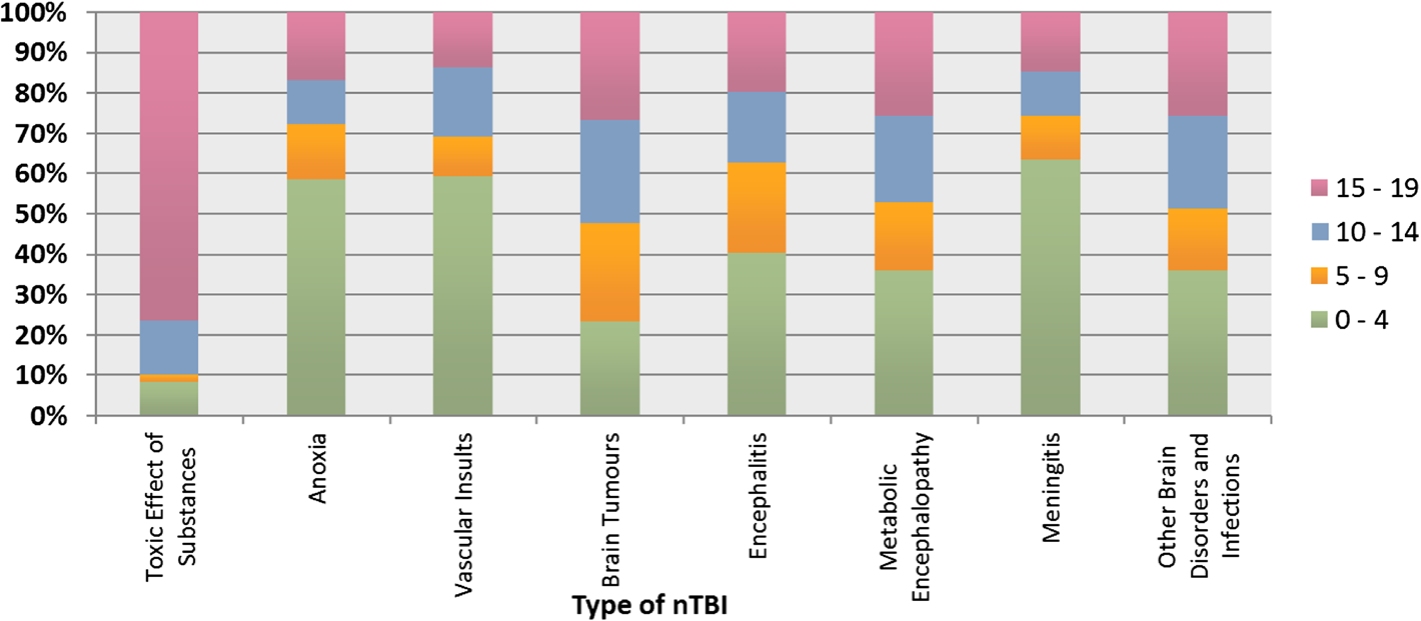
Distribution of age by type of nTBI

Across the types of nTBI diagnoses, toxic effect of substance episodes of care was found to be the highest (22.7 per 100,000), followed by brain tumour episodes of care (18.4 per 100,000), and meningitis episodes of care (15.4 per 100,000) (Table 4, Fig. 3). Patient and clinical characteristics and discharge destinations varied by type of nTBI diagnosis; 76 % of individuals presenting with toxic effects of substances were adolescents, however, 86 % of anoxic brain injury cases were infants (Fig. 4). More than half of the patients with a toxic effect of substance stayed in acute care for less than 2 days. However, 46 % of cases with vascular insult stayed in acute care for 12 days or longer. Almost 60 % of cases with vascular insult stayed in the intensive care unit for at least one day, compared to approximately a quarter of cases with encephalitis and meningitis. Overall, there was a small percentage of deaths in acute care, however, 19 % of all patients with anoxic brain injury died in acute care (Table 3).

**Table 4:** nTBI episodes of care (n and rate per 100,000 children and youth aged 19 years and under) in Ontario between fiscal years 2003/04 and 2009/10 by type of nTBI and sex

	Toxic effects of substance n rate	Anoxia n rate	Vascular insults n rate	Brain tumours n rate	Encephalitis n rate	Metabolic encephalopathy n rate	Meningitis n rate	Other brain disorders and infections n rate
Overall								
2003/04	709 22.6	293 9.4	58 1.9	605 19.3	138 4.4	106 3.4	461 14.7	333 10.6
2004/05	809 25.8	294 9.4	43 1.4	557 17.8	125 4.0	84 2.7	453 14.5	312 10.0
2005/06	742 23.7	323 10.3	60 1.9	569 18.2	109 3.5	112 3.6	475 15.2	322 10.3
2006/07	691 22.1	317 10.1	48 1.5	562 18.0	130 4.2	84 2.7	392 12.5	264 8.4
2007/08	711 22.8	251 8.1	64 2.1	492 15.8	116 3.7	86 2.8	421 13.5	272 8.7
2008/09	681 21.9	299 9.6	66 2.1	602 19.4	164 5.3	123 4.0	579 18.6	332 10.7
2009/10	625 20.1	305 9.8	42 1.4	635 20.4	181 5.8	98 3.2	585 18.8	306 9.9
2003/04 – 2009/10	4968 22.7	2082 9.5	381 1.7	4022 18.4	963 4.4	693 3.2	3366 15.4	2141 9.8
Males								
2003/04	336 21.0	172 10.7	36 2.2	364 22.7	72 4.5	50 3.1	281 17.5	188 11.7
2004/05	365 22.8	173 10.8	28 1.7	331 20.7	55 3.4	43 2.7	266 16.6	175 10.9
2005/06	351 21.9	203 12.7	33 2.1	360 22.5	59 3.7	54 3.4	255 15.9	185 11.5
2006/07	338 21.1	196 12.2	32 2.0	334 20.8	56 3.5	40 2.5	217 13.5	158 9.9
2007/08	349 21.9	151 9.5	41 2.6	257 16.1	71 4.4	46 2.9	251 15.7	147 9.2
2008/09	308 19.4	188 11.8	42 2.6	345 21.7	86 5.4	76 4.8	317 19.9	168 10.6
2009/10	273 17.2	185 11.6	29 1.8	322 20.2	97 6.1	58 3.6	327 20.6	167 10.5
2003/04 – 2009/10	2320 20.7	1268 11.3	241 2.2	2313 20.7	496 4.4	367 3.3	1914 17.1	1188 10.6
Females								
2003/04	373 24.4	121 7.9	22 1.4	241 15.8	66 4.3	56 3.7	180 11.8	145 9.5
2004/05	444 29.0	121 7.9	15 1.0	226 14.8	70 4.6	41 2.7	187 12.2	137 9.0
2005/06	391 25.6	120 7.9	27 1.8	209 13.7	50 3.3	58 3.8	220 14.4	137 9.0
2006/07	353 23.1	121 7.9	16 1.0	228 14.9	74 4.9	44 2.9	175 11.5	106 6.9
2007/08	362 23.8	100 6.6	23 1.5	235 15.5	45 3.0	40 2.6	170 11.2	125 8.2
2008/09	373 24.6	111 7.3	24 1.6	257 17.0	78 5.1	47 3.1	262 17.3	164 10.8
2009/10	352 23.2	120 7.9	13 0.9	313 20.6	84 5.5	40 2.6	258 17.0	139 9.2
2003/04 – 2009/10	2648 24.8	814 7.6	140 1.3	1709 16.0	467 4.4	326 3.1	1452 13.6	953 8.9

## Discussion

This study is the first study, to the best of our knowledge, providing a comprehensive overview of the burden of pediatric nTBI on healthcare services. Consistent with the trends seen in the overall nTBI population in Ontario [9], the number of nTBI episodes of care among children and youth increased from 2003/04 to 2009/10. However, differences were observed in discharge patterns. Of note is that the majority of children and youth were discharged home post-acute care, of which 9 % were discharged home with support services. This differs from findings on the overall nTBI population in Ontario, where less than 40 % were discharged home and 20 % died in acute care [3, 9]. This finding on discharge home is in line with studies that indicate a preference to discharge children and youth home post injury [41]. Given these differences in the healthcare use of the pediatric population compared to the general population, a specific focus on the health service use of this pediatric population is needed.

The pediatric nTBI population also differs from the TBI population; although the rate of nTBI episodes of care in this study was not as high as the rates reported for the TBI population, which ranged from 125 to 1,337 per 100,000 [34, 42, 43], the health service use of the nTBI population is just as high as the TBI population. For example, it was reported that the average LOS for a TBI related hospitalization in Canada was 5 days [44]; this paper showed that that the average LOS for a nTBI related hospitalization was approximately 13 days. Further, death in acute care for the TBI population was reported to be 9 % in Canada [44]. However, among those with anoxic brain injury, up to 19 % died in acute care. Although nTBI is not as common as TBI, this population puts an increased burden and demand on the healthcare system. Moreover, there is relatively little data suggesting how we can effectively and appropriately allocate the resources and support for these patients. Given that patients with TBI and nTBI are often treated in similar settings [13, 14] and that they use almost triple the amount of services, data pertaining specifically to the nTBI population is crucial.

Equally important is an understanding of the profile and use of healthcare services associated with each subtype of nTBI. For example, despite an overall increasing rate of nTBI episodes of care, differences were observed between types of nTBI, which may be due to the improvement in diagnosis or treatment, prevention, or coding practices. For example, there was an overall decrease in the rate of toxic effect of substances episodes of care, which may be attributed to increased prevention efforts against hazardous drinking, particularly in adolescents. Conversely, the slight increase in brain tumour episodes of care may reflect better detection of brain tumours while the increase in encephalitis may reflect trend towards a diagnosis/coding of “unspecified” or “unknown” cause of encephalitis [45–48], a diagnosis that is captured in this study. These preliminary data on subtypes of nTBI highlight the importance of stratifying the nTBI population by subtype. Also, consistent with studies on anoxic brain injury among the pediatric population [19, 20], nearly one in five patients died in acute care. Data stratified by cause of anoxic brain injury can inform the prevention of anoxic brain injuries and preparation of healthcare for this population, as it has been reported that outcomes of anoxic brain injury from a cardiac arrest vs. near drowning are significantly different [20]. Furthermore, the differences in discharge destinations across types of nTBI reflect the complexities and diversity of this group. While very few patients with a diagnosis of toxic effect of substances are discharged home with support services, more than a fifth are transferred to another inpatient facility. Centres that receive these patients, including addiction treatment centres, should be aware of and consider the effects of a brain injury on these patients when assessing treatment options. Finally, the distribution of age group across each subtype of nTBI is also varied, presenting opportunities for targeted interventions by age. These data by subtype of nTBI provide the foundation needed for further in-depth research on each type of nTBI to inform targeted resource allocation and healthcare planning.

Limitations associated with this study include a lack of consistent, agreed upon case definition for nTBI. The importance of this is highlighted in a comparison of data presented by Wong and colleagues in 2001 on non-traumatic coma. Despite similar goals on informing healthcare resources for this population, the method used to identify patients were different, resulting in higher rates of nTBI episodes of care (82.3 per 100,000) reported in this study compared to data presented by Wong and colleagues (30.8 per 100,000) [49]. Specifically, Wong and colleagues restricted participants to only those with “significantly depression of conscious level as defined by the Glasgow Coma Score of 12” and, among those under the age of 5 years, at least 6 h of unconsciousness. Conversely, this paper identified nTBI using ICD-10 codes and the codes included in this study likely captured patients with a less severe nTBI. Limitations related to the data source are also recognized. First, the DAD only captures individuals that are admitted to an acute care setting and thus, deaths that occur outside of the hospital are missed, which is considered a limitation in neurotrauma research [50]. Second, the use of all 10 diagnosis fields in the NACRS and 25 diagnosis fields in the DAD resulted in counting of all multiple nTBI diagnoses, as a patient may have more than one type of nTBI. This is an important methodological issue to consider for future studies involving statistical analyses between and/or across each type of nTBI. Third, the use of only the DAD and the NACRS to identify cases of brain tumours may result in underestimates, as brain tumours may not be readily identified or captured in the ED and/or acute care setting. The inclusion of the Ontario Cancer Registry [51], which captures information on all newly diagnosed cases of invasive neoplasia, may result in a higher number of brain tumour cases captured. As such, it is acknowledged that estimates of brain tumours in this study are likely underestimates. Nonetheless, effort to capture all potential cases that may present in the emergency department or acute care was made by using episodes of care to assess the burden of healthcare services. For example, toxic effects of substances are likely to present in the emergency department, however, this diagnosis may not be made in the acute care setting. As such, the linkage of the DAD to the NACRS helped ensure that cases that are not coded as ‘toxic effects of substances’ in the DAD were captured in the NACRS and that double counting did not occur. The data sources used in this study are also population based and Ontario has publicly funded healthcare. Therefore, data presented here are less likely to have been influenced by access supplemental health insurance. Finally, it is acknowledged that the Charlson Comorbidity Index may not be ideal for comorbidity information in the children and youth population. However, this was chosen to provide consistency for comparison with published population based data on the adult and older adult ABI populations in Ontario, Canada [3, 4, 7, 8]. It was also used to assess the severity of comorbid health conditions, which has been used previously in research looking at the pediatric population [39]. Future studies should consider exploring comorbidities through ICD-10 Chapter Headings [52] or the John Hopkins Aggregated Diagnostic Grouping (ADG) [53].

## Conclusions

To the best of our knowledge, this is the first population-based study to provide an overview of children and youth with nTBI in Ontario, Canada. Findings from this study provide a foundation for further in-depth research on nTBI and its subtypes that are critical for resource allocation and support for children and youth with nTBI. The availability of accurate and timely information on nTBI is crucial for the planning of healthcare services, resource allocation, and prevention. Children and youth are at a critical developmental period of their lives in which a nTBI can result in negative and lasting consequences. Some types of nTBI identified in this study are preventable; a focus on nTBI and in-depth research on each subtypes of nTBI is encouraged.

## Abbreviations

ABI, acquired brain injury; CCC, complex continuing care; CIHI, Canadian Institute for Health Information; DAD, Discharge Abstract Database; ICD-10, International Classification of Diseases Version 10; LOS, length of stay; LTC, long-term care; NACRS, National Ambulatory Care Reporting System; nTBI. non-traumatic brain injury; TBI, traumatic brain injury
